# Extended pelvic lymph node dissection during robotic prostatectomy: antegrade versus retrograde technique

**DOI:** 10.1186/s12894-024-01448-1

**Published:** 2024-03-21

**Authors:** Giancarlo Albo, Andrea Gallioli, Francesco Ripa, Elisa De Lorenzis, Luca Boeri, Carolina Bebi, Lorenzo Rocchini, Fabrizio Longo, Stefano Paolo Zanetti, Matteo Turetti, Michela Piccoli, Emanuele Montanari

**Affiliations:** 1 Department of Clinical Sciences and Community Health, University of Milan, Milan, Italy; 2 Department of Urology, IRCCS Foundation Ca’ Granda, Ospedale Maggiore Policlinico, Milan, Italy; 3 Department of Urology, Fundació Puigvert, Barcelona, Spain; 4 Department of Urology, Whittington Health NHS Trust, London, UK; 5 Department of Paediatric Urology, Great Ormond Street Hospital for Children, London, WC1N 3JH UK; 6 Cantonal Hospital Entity Regional Hospital of Bellinzona and Valleys (ORBV) A., Gallino Street 12, CH-6500 Bellinzona, Switzerland

**Keywords:** Prostatectomy, Lymphadenectomy, Minimally Invasive Surgery, Prostate Cancer, Robotics, Complications

## Abstract

**Background:**

Robot-assisted radical prostatectomy (RARP) with extended lymphadenectomy (ePLND) is the gold standard for surgical treatment of prostate cancer (PCa). Recently, the en-bloc ePLND has been proposed but no studies reported on the standardization of the technique. The aim of the study is to describe different standardized en-bloc ePLND, the antegrade and the retrograde ePLND, and to compare their surgical and oncological outcomes.

**Materials & Methods:**

From January 2018 to September 2019, all patients subjected to RARP plus ePLND by one single surgeon were enrolled. ePLND was performed in a retrograde fashion by starting laterally to the medial umbilical ligament from the internal inguinal ring proceeding towards the ureter, or in an antegrade way by starting from the ureter at its crossing with the common iliac artery and proceeding towards the femoral canal. Patients’ demographic data, clinical and surgical data were collected. Each en-bloc ePLND was categorized as “efficient” or “inefficient” by the operator, as surrogate of surgeon’s satisfaction.

**Results:**

Antegrade and retrograde ePLND were performed in 41/105 (group A) and 64/105 (group R) patients, respectively. The two groups (A vs R) had similar median (IQR) number of lymph nodes retrieved [20 (16.25–31.5) vs 19 (15–26.25); *p* = 0.18], ePLND time [33.5 (29.5–38.5) min vs 33.5 (26.5–37.5) min; *p* = 0.4] and post-operative complications [8/41 (19.5%) vs 9/64 (14.1%); *p* = 0.61]. In group A, 3/41 (7.3%) clinically significant lymphoceles were reported, while 1/64 (1.6%) in group R (*p* = 0.3). 33/41 (80.5%) and 28/64 (44%) procedures were scored as efficient 59 in group A and R, respectively (*p* = 0.01). On multivariate regression, only BMI (B = 0.93; 95% CI 0.29–1.56; *p* = 0.005) was associated with a longer ePLND time.

**Conclusions:**

The study indicates that antegrade and retrograde en-bloc extended pelvic lymph node dissection (ePLND) have comparable surgical and oncologic outcomes, supporting the importance of standardizing the procedure rather than focusing on the direction. Although both techniques aligned with current evidence regarding lymph node invasion and complications, the antegrade approach was subjectively perceived as safer due to early isolation of critical anatomical landmarks. Encouragement for the use of en-bloc ePLND, regardless of direction, is emphasized to improve prostate cancer staging accuracy and procedural standardization.

**Supplementary Information:**

The online version contains supplementary material available at 10.1186/s12894-024-01448-1.

## Introduction

Prostate cancer (PCa) is the second most commonly diagnosed non-cutaneous tumor in males wordwide [1]. The introduction of systematic PSA-screening has substantially increased the detection of localized disease. Robot-assisted radical prostatectomy (RARP) with (or without) lymphadenectomy is the gold-standard for surgical treatment for PCa [2]. Pelvic lymph node dissection (PLND) during radical prostatectomy provides important information for both tumour staging and patients’ prognosis [3].

The lymphatic drainage of the prostate, as determined by lymphography, [4] spreads into 3 main directions to reach the ipsilateral lymph nodes. The lymphatics from the superolateral angle of the prostate travel along the lateral pelvic wall to drain to the internal iliac lymph nodes. Some lymphatics also drain posteriorly to reach the presacral lymph nodes and, in 45% of patients, lymphatics form the apex of the prostate drain along the internal iliac artery to reach again the internal iliac lymph nodes. The nodal drainage of the prostate can thus vary among individuals and can be undersampled by limited template dissection.

**Fig. 1:**
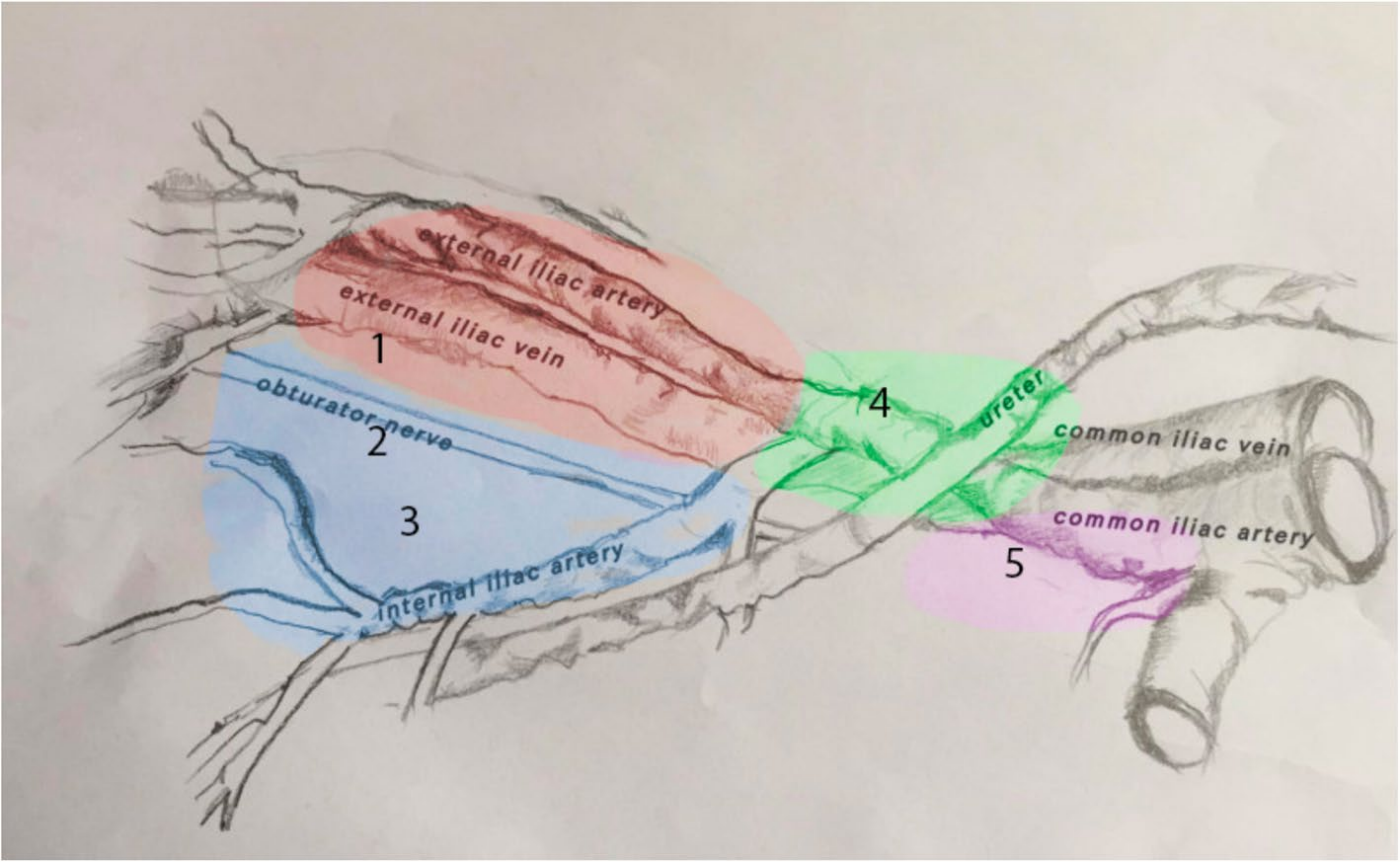
Anatomic limits of pelvic lymph node dissection (PLND). Area 1 (red): limited PLND. Areas 1–3 (blue): standard PLND. Areas 1–4 (green): extended PLND. Areas 1–5 (purple): superextended PLND

Boundaries and nomenclature for lymph node dissection were first described for bladder cancer [5] and later confirmed for prostate cancer [6]. The extended lymphadenectomy template includes all the areas mentioned above, from the midportion of the common iliac arteries to the inguinal ligament, including the nodes in the obturator region and the presciatic fossa of Marceille’s (Fig. 1). Several studies compared the extended pelvic lymph node dissection (ePLND) with standard PLND (sPLND) and demonstrated that the ePLND provides a better staging of the disease. Nevertheless, the therapeutic effect of lymphadenectomy is yet to be determined [3]. When the ePLND is performed during RARP, it yields similar results as seen in open and laparoscopic procedures in terms of number of nodes removed, likelihood of node positivity and types and rates of complications [6]. Unlike the robotic prostatectomy procedure, which is highly standardized in each of its surgical steps, lymphadenectomy is not. The literature indeed defines the anatomical area of interest, but it lacks standardized descriptions of the surgical technique. As of today, the most standardized description of iliac lymphadenectomy during robotic prostatectomy is the mono-block robotic ePLND, which allows for the removal of the lymph nodes in the iliac area as a single tissue block [7]. The procedure can either be performed in a retrograde fashion by starting laterally to the medial umbilical ligament at the entrance of the femoral canal and proceeding towards the ureter, or in an antegrade fashion by starting from the ureter at its crossing with the common iliac artery and proceeding towards the femoral canal [7]. To date, no study has ever compared the two techniques. Therefore, the aim of this study is to standardize these different approaches and to explore the surgical and oncological outcomes of antegrade and retrograde ePLND.

## Materials & Methods

### Study design

After obtaining approval from the Institutional Review Board (n 0029354), a retrospective analysis was conducted on all patients who underwent RARP with ePLND between January 2018 and September 2019 in our academic center. According to NCCN guidelines on Prostate cancer, the indication for ePLND was the probability of nodal metastases ≥ 2% based on preoperative characteristics [8]. All procedures were performed by a single experienced surgeon (G.A.). The retrograde technique, which represented the standard approach for the surgeon, was used from January 2018 to December 2018, whereas from January 2019 onwards, the antegrade or retrograde approach were applied based on the surgeon's preference and patient’s anatomy. During the surgical procedure the chosen technique (antegrade vs retrograde dissection) was applied to both the left and right sides of the dissection for each patient, ensuring a consistent approach within the same individual. Previous abdominal surgical interventions may have led to the presence of intestinal adhesions, making exposure of the iliac area more challenging. However, this condition did not act as an exclusion factor: in all cases where lymphadenectomy was indicated, the procedure was still performed Peri-operative variables included: demographic data, preoperative PSA (iPSA), clinical and pathological stage of the disease (according to 2017 TNM classification) [9], ISUP grade, number of resected lymph nodes and percentage of positive ones, rate of overall perioperative complications, overall operating time (OT), estimated blood loss (EBL), complications at 30 days according to the Clavien-Dindo classification [10], pathological staging, length of hospital stay and total days of catheterization. In order to measure the surgeon’s overall satisfaction in terms of ability to follow the surgical steps and to perform an *en-bloc* dissection, each procedure was classified as either "efficient" or "inefficient," disregarding the dissection direction. The categorization depended on criteria, including the absence of bleeding, clear identification of anatomical landmarks, and the prevention of iatrogenic injuries to surrounding anatomical structures.

Complications such as fever and lymphocele formation were reported in medical reports, outpatient evaluations during follow-up and accesses to the emergency department. Lymphoceles were defined as clinically significant in case of fever, monolateral leg oedema, compression of the iliac vessels (demonstrated by eco-color doppler ultrasound) or visible mass on physical examination.

Follow up included cystography 6 days after RARP, blood exam 15 days after the discharge and PSA dosage at 45 days. No systematic pelvic ultrasound was performed during follow up in absence of symptoms.

The study was conducted in accordance with the Declaration of Helsinki (1964) and its later amendments. All patients signed an informed consent at the time of hospitalization to share their clinical information anonymously for research purposes. Data were prospectively collected in an encrypted multivariable database and were analysed retrospectively.

### Surgical technique

All RARPs were performed with Da Vinci Si (Intuitive Surgical, Sunnyvale, CA, USA) with a transperitoneal approach. Patients were operated in the lithotomy position, as described by Menon [11]. The Trendelenburg was set at 25° and the robot was positioned between the legs of the patient for docking. All RARPs were performed according to Patel’s technique [12]. The ePLND was performed before the prostatectomy. The following instruments were employed for ePLND: monopolar scissors on arm 1, bipolar maryland on arm 2, and prograsp on arm 3.

Cefazoline 2 g was administrated for antibiotic prophylaxis.

After the surgery, a 18 ch percutaneous abdominal drain was positioned, and usually removed on postoperative day 1. Patients were usually discharged on postoperative day 2.

### Antegrade en-bloc ePLND

**Fig. 2:**
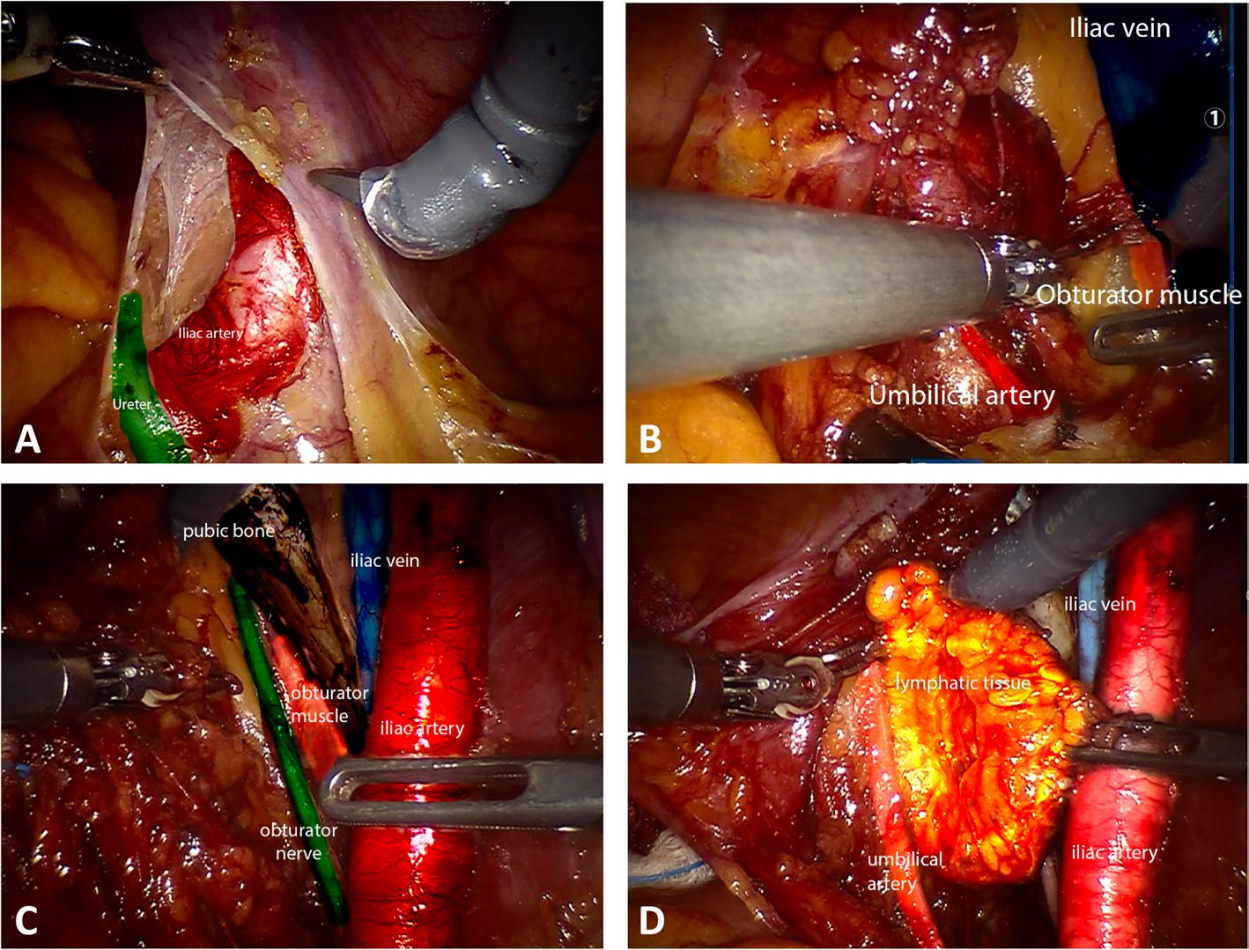
**A**. The peritoneum is incised where the ureter crosses the common iliac artery exposing both the artery and the ureter. **B**. Exposition of iliac vein, umbilical artery at its origin, and of Marceille’s triangle in antegrade fashion. **C**. Exposition of obturator nerve in obturator fossa. **D**. enbloc antegrade lymph node dissection is completed. The lymphatic tissue is isolated from umbilical artery

In the antegrade *en-bloc* technique, the peritoneum is incised where the ureter crosses the common iliac artery (Fig. 2A). Both the artery and the ureter are then exposed. The lymph node dissection starts on the common iliac artery at the level of the ureter’s crossing and it proceeds in an antegrade direction, until the femoral canal and the vas deferent are exposed. The dissection continues further deeply exposing the common iliac vein right under the ureter and proceeds again in an antegrade fashion down to the femoral canal. The hypogastric artery, which is the medial border of the surgical field, is isolated, and the umbilical obliterated artery is exposed at its origin (Fig. 2B). Access to the Marceille’s triangle is developed in antegrade fashion starting from the medial border of the psoas major. The obturator nerve is then identified (Fig. 2C). The isolated lymphatic tissue is pulled anteriorly and released in the obturator fossa at the level of the iliac vessels. The vas deferens is then identified and divided. The lymphatic tissue over the obturator nerve is resected with a combined blunt and sharp dissection. The umbilical artery is followed, and the lymphatic tissue is divided in antegrade direction (Fig. 2D). The *en-bloc* antegrade lymph node dissection is then completed.

### Retrograde en-bloc ePLND

**Fig. 3:**
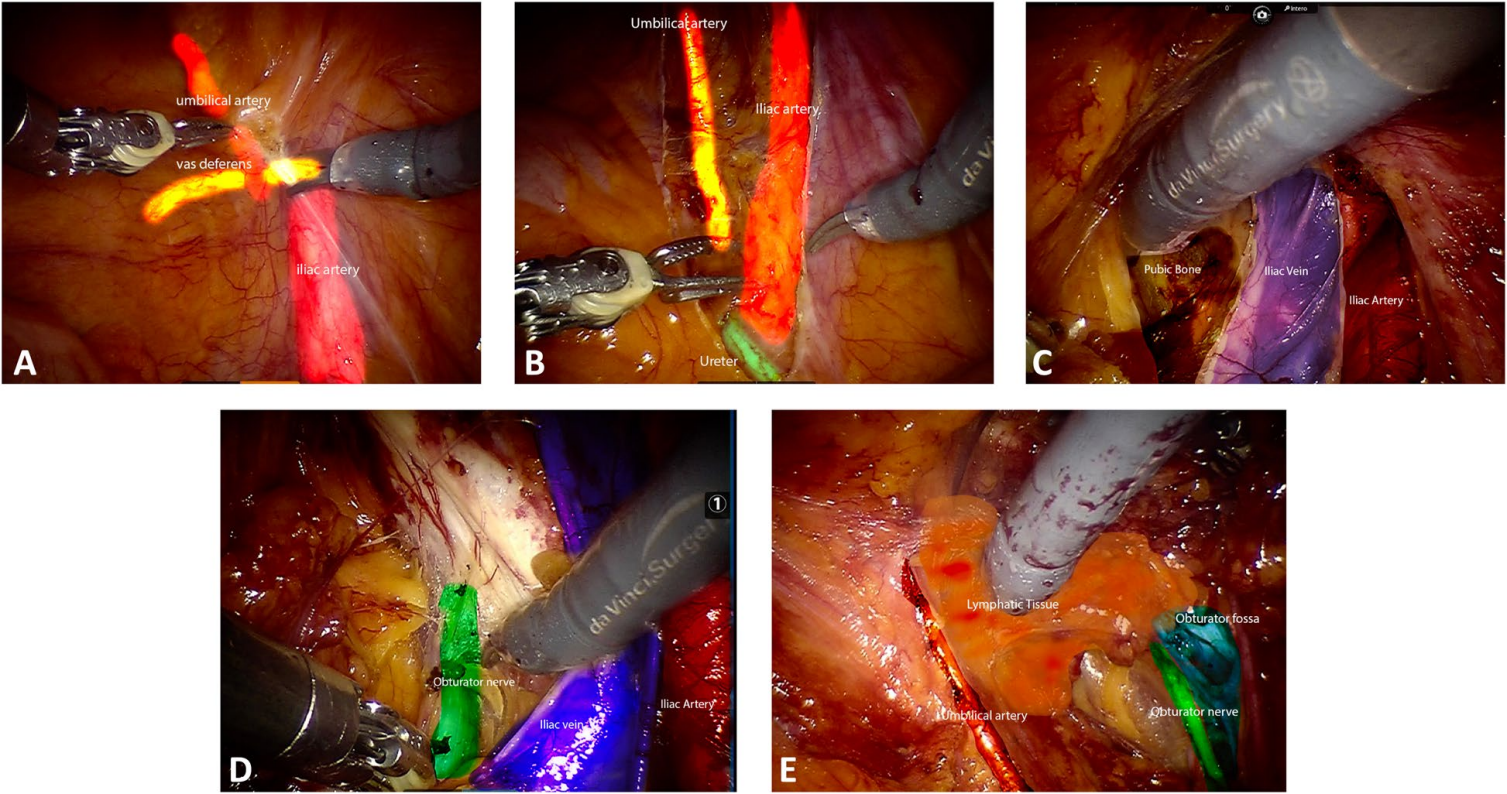
**A**. The peritoneum is incised at the level of the external iliac artery, at the level of inferior epigastric vessels, medial to the spermatic cord. **B**. The incision advances in a retrograde direction, until the ureter is identified. **C**. The lymph node dissection starts on the external iliac vessels and proceeds in a retrograde fashion until the ureter. **D**. The obturator nerve is identified distally at the level of obturator foramen. **E**. The enbloc retrograde lymph node dissection is completed by detaching the lymphatic tissue from the umbilical artery

In the retrograde technique the peritoneum is incised at the level of the external iliac artery, at the level of inferior epigastric vessels, medial to the spermatic cord (Fig. 3A). The incision advances in a retrograde direction, until the ureter is identified (Fig. 3B). The lymph node dissection starts on the external iliac artery close to the vas deferent. The vas deferent is then divided and the dissection continues distally, in the femoral canal, until the external iliac vein is exposed. The external and the common iliac arteries are exposed up to the ureter, as for the iliac vein, with a combined blunt and sharp dissection (Fig. 3C). After the iliac vessels are completed exposed, the dissection proceeds in the obturator fossa. The obturator nerve is identified distally at the level of obturator foramen (Fig. 3D). The access to the Marceille’s triangle is achieved in a retrograde fashion, in the same fashion as in the antegrade dissection, towards the medial border of psoas major, exposing the obturator nerve both medially and laterally. The *en-bloc* retrograde lymph node dissection is completed by detaching the lymphatic tissue from the umbilical artery (Fig. 3E).

### Statistical analysis

Distribution of data was tested with the Shapiro–Wilk test. Data are presented as medians (interquartile range; IQR) or frequencies (proportions). Demographics characteristics, intraoperative and postoperative data were compared between antegrade (Group A) and retrograde (Group R) ePLND procedures with the Mann–Whitney test and the Fisher exact test. Multivariable (MVA) linear regression analysis were used to identify variables (e.g., previous abdominal surgery, technique of *en-bloc* ePLND, lymphadenomegaly, BMI) associated with ePLND time. Likewise, MVA logistic regression analysis tested the association between the same variables and the surgeon’s satisfaction of the ePLND.

Statistical analyses were performed using SPSS v.26 (IBM Corp., Armonk, NY, USA). All tests were two sided, and statistical significance level was determined at *p* < 0.05.

## Results

Table 1 depicts descriptive statistics of the entire cohort of participants as segregated according to the type of ePLND. Antegrade and retrograde lymph node dissection were performed in 41 (39%) and 64 (61%) patients, respectively. Overall, age, BMI, iPSA, clinical stage, ASA score and rates of previous abdominal surgery were comparable between groups.

**Table 1:** Descriptive statistics of the entire cohort of participants as segregated according to the type of LND

	**ePLND during RARP**		
	**Antegrade**	**Retrograde**	***p*** value*
**No. Patients**	41	64	
**Age (years)**	67 (IQR 62–72.5)	70 (IQR 64–72)	0.27
**BMI (kg/m**^**2**^**)**	25.95 (IQR 24.22–29.76)	25.45 (IQR 23.53–28.36)	0.45
**ASA**			0.34
**I**	15 (36.6%)	29 (45.3%)	
**II**	23 (56.1%)	32 (50.0%)	
**III**	3 (7.3%)	3 (4.7%)	
**Previous abd. surgery**			0.22
**Yes**	23 (56.1%)	28 (43.8%)	
**No**	18 (43.9%)	36 (56.3%)	
**Clinical Stage**			
**cT1**	20 (48.8%)	30 (46.9%)	0.78
**cT2**	21 (51.2%)	32 (50.0%)	0.78
**cN1**	1 (2.4%)	4 (6.3%)	0.37
**iPSA**	7.35 (IQR 5.12–10.35)	6.95 (IQR 4.92–10)	0.97
**ISUP grade**			0.99
**1**	4 (9.8%)	6 (9.4%)	
**2–3**	24 (58.5%)	42 (65.6%)	
**4–5**	13 (31.7%)	16 (25%)	
**Pathological stage**			0.31
**pT2b-pT2c**	28 (68.3%)	40 (62.5%)	
**pT3a**	6 (14.6%)	11 (17.2%)	
**pT3b**	4 (9.7%)	5 (7.8%)	
**Lymph nodes**			
**Total number**	20 (IQR 16.25–31.5)	19 (IQR 15–26.25)	0.18
**Positive lymph nodes**	5/41 (12.2%)	8/64 (12.5%)	0.96
**Total OT (min)**	290 (IQR 262–305)	326 (IQR 301.25–347.5)	< 0.001
**ePLND time (min)**	33.5 (IQR 29.5–38.5)	33.5 (IQR 26.5–37.5)	0.40
**EBL (ml)**	300 (IQR 195–417.5)	323 (IQR 200–483)	0.25
**Length of stay (days)**	2.50 (IQR 2–3)	2.50 (IQR 2–3)	0.93
**Catheterisation (days)**	7 (IQR 6–9)	7 (IQR 6–7.75)	0.71
**Abdominal drain (days)**	1 (IQR 1–1)	1 (IQR 1–1)	0.56
**Complications**			0.61
**Yes**	8 (19.5%)	9 (14.1%)	
**No**	33 (80.5%)	55 (85.9%)	
**Clavien-Dindo**			0.40
**I-II**	6 (14.6%)	9 (14%)	
**III-IV**	2 (4.9%)	0 (0%)	
**Lymphocele**			0.30
**Yes**	3 (7.3%)	1 (1.6%)	
**No**	38 (92.7%)	63 (98.4%)	
**Fever**			0.51
**Yes**	5 (12.2%)	5 (7.8%)	
**No**	36 (87.8%)	59 (92.2%)	
**LND quality**			
**Efficient**	33 (80.5%)	28 (44%)	0.01
**Not efficient**	8 (19.5%)	36 (56%)	

Keys: *ePLND*  extended pelvic lymph node dissection, *RARP*  Robot-assisted radical prostatectomy, *BMI*   Body mass index, *ASA* American Society of Anesthesiologist classification, *EBL* Estimated blood loss

^*^p value according to the Mann–Whitney U test and the Fisher exact test, as indicated

In total, 51 previous abdominal surgeries were performed, including 26 inguinal hernia repairs, 22 appendectomies, 5 cholecystectomies, 2 endoscopic removals of rectal polyps, 1 gastric banding, and 1 umbilical hernia repair. Nine patients underwent more than one abdominal surgery.

Pathological stage of the two groups were similar. The median (IQR) number of total lymph nodes retrieved in group A was 20 (16.25–31.5), while for group R it was 19 (15–26.25) (*p* = 0.18). Besides, the rate of positive lymph nodes (with evidence of neoplastic dissemination) was 5/41 (12.2%) for group A vs 8/64 (12.5%) for group R (*p* = 0.96). Total OT was higher in Group R than Group A (*p* < 0.001) but the time of ePLND was similar among groups. EBL, LOS, timing of abdominal drainage removal and days of catheterization were similar between groups. Overall, 17/105 (16.2%) patients had post-operative complications, with no difference between the two groups (*p* = 0.61). According to the Clavien-Dindo classification, 15 patients reported Clavien I or II complications: among these, 6 belonged to group A and 9 belonged to group R (*p* = 0.4). The 2 patients who presented with Clavien IIIa complications had an antegrade ePLND. Specifically, the first one developed bilateral hydronephrosis that required positioning of DJ stents for renal decompression; the second one presented with a clinical lymphocele necessitating of percutaneous drainage. Overall, we reported 4/105 (3.8%) cases of lymphocele, but their distribution was comparable between the two techniques (*p* = 0.30). Similar results were found concerning the rates of post-operative fever, with 5/41 (12.2%) among the antegrade and 5/64 (7.8%) among the retrograde procedures (*p* = 0.51).

As for surgeon’s satisfaction, 33/41 procedures (80.5%) in group A were scored as efficient, while 28/64 (44%) were considered efficient in group R (*p* = 0.01).

Table 2 reports linear regression analysis showing the association between clinical variables and ePLND operative time. Multivariate analysis revealed that increased BMI was associated with a longer time of lymphadenectomy (B = 0.93; 95% CI 0.29–1.56; *p* = 0.005), after adjusting for previous abdominal surgery technique of *en-bloc* ePLND.

**Table 2:** Univariate and multivariate linear regression analysis testing the association between variables and time of lymphadenectomy

	**Univariate**			**Multivariate**		
	B	95% CI	P value	B	95% CI	*P* value
**BMI**	0.70	(0.12–1.28)	0.19	0.93	(0.29–1.56)	0.005
**Previous abdominal surgery**	-1.21	(-5–69-3.26)	0.59	3.28	(-1.56–8.13)	0.18
**Antegrade vs retrograde ePLND**	0.21	(-4–39-4.81)	0.93	-0.09	(-5.07–4.89)	0.97

Keys: *ePLND*  extended pelvic lymph node dissection, *BMI* Body mass index

Table 3 depicts logistic regression analysis predicting surgeon’s satisfaction with the ePLND procedure. MVA logistic regression analysis revealed that lower BMI (OR = 0.84; *p* = 0.01) and the antegrade technique of ePLND (OR = 3.85; *p* = 0.016) were associated with increased surgeon’s satisfaction with ePLND, after accounting for previous abdominal surgery.

**Table 3:** Univariate and multivariate logistic regression for surgeon’s satisfaction of enbloc extended pelvic lymph node dissection during robot-assisted radical prostatectomy

	**Univariate**			**Multivariate**		
	OR	95% CI	P value	OR	95% CI	*P* value
**BMI**	0.84	(0.74–0.96)	0.01	0.84	(0.73–0.97)	0.01
**Previous abdominal surgery**	1.27	(0.49–3.29)	0.62	0.92	(0.3–2.8)	0.88
**Antegrade vs retrograde ePLND**	3.2	(1.19–8.60)	0.02	3.85	(1.29–11.56)	0.016

Keys: *ePLND* extended pelvic lymph node dissection, *BMI* Body mass index

## Discussion

In this study, we assessed the surgical outcomes associated with retrograde versus antegrade en-bloc ePLND during RARP. Significantly, both approaches demonstrated comparable results in terms of surgical outcomes and the number of retrieved lymph nodes.

The standardization of the lymphadenectomy guarantees removal of a reproducible number of lymph nodes from the surgical field, irrespectively from the direction of the approach. The importance of surgical procedure standardization is well recognized, as it leads to shortening of learning curves through the repetition of technical movements. In fact, RARP has been divided in multiple steps that form the modular robotic training curriculum of the European Association of Urology [13]. In addition, the surgical steps performed by the bedside assistant during RARP have also been standardized [14]. The same concept is yet to be applied for lymphadenectomy, which steps are usually skipped also during live-surgeries [15]. Literature is mainly focused on anatomical boundaries of PLND and on the extension of lymphadenectomy, from standard to extended and super extended, during mini-invasive abdominal surgery [16, 17]. Although the therapeutic effect of lymphadenectomy still has to be clarified [3], it has been demonstrated that surgical volume impacts on the number of lymph nodes removed and rates of lymph node invasion [18]. Moreover, several studies reported a lower number of lymph nodes excised in mini-invasive prostatectomy compared to the open technique [19]. This may be due to the absence of a standardization of the procedure. Mattei et al. demonstrated that the use of “monoblock” ePLND could be useful to guarantee the excision of a template of lymph nodes similar to that used in the open lymphadenectomy and standardised the technique for both the extended and superextended PLND [7, 17]. By this way the surgeon can better respect the PLND template and correctly define the type of lymphadenectomy that has been performed. Moreover, the systematic presentation of the specimen (en-bloc versus separate package) might influence the histological report, as already demonstrated for bladder cancer [20]. As a matter of fact, the heterogenous processing of specimens still represents a major concern, and currently has a significant role in the staging of PCa [21].

To date, this is the first study that compares antegrade *en-bloc* ePLND to retrograde *en-bloc* ePLND during RARP, providing an educational video. During ePLND is essential identify and isolate the anatomical landmarks that are crucial to perform the lymphadenectomy at the required extension, i.e. the ureter, the Marceille’s triangle, and the hypogastric artery (with the origin of the umbilical artery). The direction of the procedure, whether retrograde or antegrade, involves different control of anatomical landmarks. In the case of a retrograde procedure, the initial structures identified are at the level of the internal inguinal ring. These structures include the vas deferens, gonadal vessels, external iliac artery, and inferior epigastric vessels. On the other hand, in the case of an antegrade procedure, the first structures identified are at the cranial limit of the lymphadenectomy: the ureter, common iliac vessels, iliac bifurcation, and the obliterated umbilical artery at its emergence from the hypogastric artery.

In this study, we initially standardized the two approaches and then aimed to assess if they are comparable in terms of clinical outcomes. We found that the OT of ePLND was comparable between the two approaches, thus it could not be the only variable accountable for the different total operating time. The only variable associated with longer time of lymphadenectomy was increased BMI. Lastly, we focused on two paradigmatic complications of RARP and LND: post-operative fever and lymphocele occurring within 30 days from surgery. We only observed a few cases of symptomatic lymphocele (4) and fever (10) but, as reported above, they were equally likely to happen in the antegrade and in the retrograde groups. Additionally, this result is in line with the current evidence, that shows a high number of asymptomatic lymphoceles (51% of cases) [22] and a variable number of clinically significant lymphoceles (3–7.9%) [3, 22].

We also tried to highlight the different perception of the operating surgeon in performing antegrade and retrograde dissection and we showed a preference for the antegrade approach, with higher rates of subjective satisfaction and comfort, even after adjusting for possible confounding factors.

The definition of efficiency, however, is highly subjective. The procedure was classified as efficient or non-efficient based on whether it was bloodless, if anatomical landmarks were clearly identified, and if there were no iatrogenic injuries to surrounding anatomical structures. The definition of efficiency was binary, thus the procedure was simply evaluated as either efficient or non-efficient. The perceived advantage of the antegrade technique is the possibility to identify the above-mentioned landmarks as the first step. The dissection of the lymph node package proceeds having all the critical structures on sight which is perceived as safer by the surgeon. In fact, the surgeon starts the lymphadenectomy respecting one of the golden rules of surgery: “from where you see to where you do not see”. This advantage is particularly evident if the ePLND is performed before RARP, while it may be less important when the lymphadenectomy is done after RARP. The incision of the peritoneum performed during RARP changes the relationships between the anatomical landmarks, mainly at the level of origin of the umbilical artery on the hypogastric vessel which represents the medial border of the dissection. Instead, the exposition of pubis, and of the obturator fossa previously gained during prostatectomy makes the retrograde approach easier. Moreover, the retrograde approach may be advisable in the presence of intestinal-peritoneal adhesions. Indeed, these adhesions can hinder the identification of the ureter (and the Marceille’s triangle) which may be safely isolated with a peritoneum incision in correspondence with the vas deferens, following the external iliac artery until the common iliac artery.

This study is not devoid of limitations. Due to its retrospective nature, no randomization was performed. RARPs were done by one single surgeon, which may rise some issues on the reproducibility of these results. Besides these critical points, the study brings to light the need for a common technique of ePLND that guarantees appropriate staging of PCa.

## Conclusion

It is crucial to highlight that the comparison between retrograde and antegrade lymphadenectomy revealed overlapping outcomes, underscoring the necessity for a prudent interpretation of the data. The current study indicates that the surgical and oncologic outcomes of antegrade versus retrograde en-bloc ePLND are comparable, emphasizing that the direction of the procedure may not be significant; rather, its standardization emerges as a key factor in achieving consistent results.

The rates of lymph node invasion and complications were in line with current published evidence for both approaches. Although both procedures were comparable, the antegrade technique was subjectively perceived as safer due to the isolation of critical anatomical landmarks as the first step of the procedure. The use of *en-bloc* ePLND, either in an antegrade or retrograde direction, should be encouraged to standardize the procedure and increase the accuracy of PCa staging.

### Supplementary Information

**Supplementary Material 1. **

## Data Availability

No research data outside the submitted manuscript are available.
