# Correction: Extended pelvic lymph node dissection during robotic prostatectomy: antegrade versus retrograde technique

**DOI:** 10.1186/s12894-024-01477-w

**Published:** 2024-04-15

**Authors:** Giancarlo Albo, Andrea Gallioli, Francesco Ripa, Elisa De Lorenzis, Luca Boeri, Carolina Bebi, Lorenzo Rocchini, Fabrizio Longo, Stefano Paolo Zanetti, Matteo Turetti, Michela Piccoli, Emanuele Montanari

**Affiliations:** 1 Department of Clinical Sciences and Community Health, University of Milan, Milan, Italy; 2 Department of Urology, IRCCS Foundation Ca? Granda, Ospedale Maggiore Policlinico, Milan, Italy; 3 Department of Urology, Fundaci? Puigvert, Barcelona, Spain; 4 Department of Urology, Whittington Health NHS Trust, London, UK; 5 Department of Paediatric Urology, Great Ormond Street Hospital for Children, London, WC1N 3JH UK; 6 Cantonal Hospital Entity Regional Hospital of Bellinzona and Valleys (ORBV) A., Gallino Street 12, CH-6500 Bellinzona, Switzerland

**Correction: BMC Urology (2024) 24:64**

10.1186/s12894-024-01448-1

Following publication of the original article [1], the author noticed an error in Funding acknowledgement. The statement in the Funding information section was incorrectly given as ‘No funding was received for conducting this study’ and should have read ‘This study was partially funded by the Italian Ministry of Health - Current Research IRCCS’.

The original article has been corrected.
